# Co-occurrence of Pigmented Villonodular Synovitis and Patellar Clunk Syndrome Following Total Knee Arthroplasty: A Case Report

**DOI:** 10.7759/cureus.73207

**Published:** 2024-11-07

**Authors:** José L Ayala-Ortiz, Chase Hobbs, John Murphy, Sameer Naranje

**Affiliations:** 1 Orthopaedic Surgery, The University of Alabama at Birmingham, Birmingham, USA

**Keywords:** diffuse pvns, knee, patellar clunk, pigmented villonodular synovitis, total knee arthroplasty (tka)

## Abstract

Pigmented villonodular synovitis (PVNS) is an uncommon hyperproliferative disease of the synovium presenting either as localized or a more aggressive diffuse form. Its occurrence following total knee arthroplasty (TKA) is rare, and its presentation alongside patellar clunk syndrome (PCS) has not been previously reported. We present a case of a 64-year-old female patient diagnosed with diffuse PVNS (D-PVNS) two and half years following TKA, co-occurring with PCS. The diagnosis was initially suspected during a diagnostic arthroscopy for PCS, after which the patient underwent arthroscopic synovectomy and scar excision. Histological analysis of the resected tissue confirmed the diagnosis, revealing inflammatory cells and hemosiderin deposits.

## Introduction

Pigmented villonodular synovitis (PVNS) is a benign yet locally invasive, proliferative disease of the synovium. It most commonly presents between the second and fifth decades of life, though it can occur at any age [1,2]. Histological findings in PVNS typically include hemosiderin depositions, lipid-laden cells, multinucleated giant cells, and inflammatory infiltrates [2]. Chromosomal alterations, particularly translocations of 11p11-13, have been implicated in PVNS, leading to the overexpression of colony-stimulating factor 1 (CSF1), which drives inflammatory responses in macrophage lineage cells [3,4].

PVNS is classified into two subtypes: localized (L-PVNS) and diffuse (D-PVNS), with presentations either intra-articular or extra-articular [3]. While PVNS can affect various joints, the knee is frequently involved, with 64% of the cases in this location being the diffuse type and 46% of the localized type [3]. Recent data indicate that L-PVNS affects 30.3 patients per one million, whereas the diffuse type affects 8.4 per one million [5]. L-PVNS typically manifests as a single pedunculated lesion in otherwise healthy synovial tissue, often in the anteromedial compartment of the knee. In contrast, the more aggressive D-PVNS involves the entire synovial lining and may cause bony erosions if left untreated. Patients with D-PVNS commonly present with pain, swelling, stiffness, and limited range of motion (ROM) [1,6-8].

Patellar clunk syndrome (PCS) is a separate complication that can arise following total knee arthroplasty (TKA), most often associated with posterior-stabilized implant designs. Patients with PCS experience symptoms ranging from painless catching of the knee to painful locking. PCS occurs due to the buildup of scar tissue along the quadriceps tendon and the patella, and its etiology is believed to be multifactorial [9].

In this report, we describe a case of PVNS diagnosed two and a half years after TKA, occurring concomitantly with PCS in a patient with no known history of PVNS. While PVNS has been reported after TKA in other case reports [10-16], diagnosing PVNS through arthroscopy in the clinical context of PCS is, to our knowledge, unique.

## Case presentation

A 64-year-old African American female patient with a past medical history of type two diabetes mellitus, hypertension, hyperlipidemia, coronary artery disease, and chronic back pain presented with a nine-month history of right knee swelling, painful ROM, and crepitus 28 months after primary TKA.

At the age of 62, the patient had initially presented to the orthopedic clinic at our institution with a five-year history of progressive right knee pain and mechanical symptoms, including several falls that interfered with her daily activities. Physical examination and radiographic findings were consistent with tri-compartmental degenerative joint disease (DJD). After failing conservative management, including physical therapy, corticosteroid injections, and pain medications, the decision was made to proceed with surgical intervention. The patient underwent a right cemented TKA with the posterior-stabilized Zimmer Biomet Persona Arthroplasty System (Warsaw, IN). The surgery was successful, with no perioperative or postoperative complications. No abnormal synovial tissue was observed during the procedure, and the patient had an uneventful recovery, being discharged with a two-week follow-up appointment.

In the months following surgery, during physical therapy and follow-up, the patient reported a fall due to her knee giving out, though she denied direct trauma to the knee. Nevertheless, she progressed well with physical therapy, achieving full ROM and strength, and resumed her daily activities comfortably.

**Figure 1:**
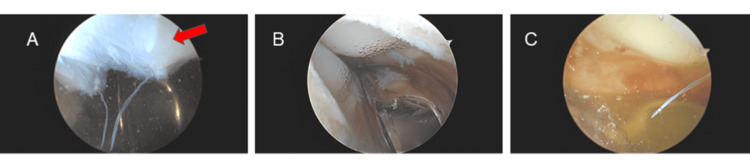
(A-C) Initial arthroscopy images showing patellar clunk syndrome (red arrow) and no signs of synovitis.

However, approximately 11 months after TKA, she returned to the clinic complaining of moderate, painful ROM and "clicking" in the right knee. She denied recent falls, trauma, or systemic symptoms. On examination, knee swelling and crepitus with ROM were noted. The incision was well-healed, and there were no signs of infection. A diagnosis of PCS was suspected, and the patient was indicated for diagnostic arthroscopy. Arthroscopy revealed a cyclops lesion and scar tissue, consistent with PCS, which were successfully excised. Intraoperative images (Figure 1) showed no hemarthrosis or synovitis. The TKA components were stable, with no evidence of loosening or polyethylene wear. Postoperatively, the patient achieved good ROM and was pain-free.

**Figure 2:**
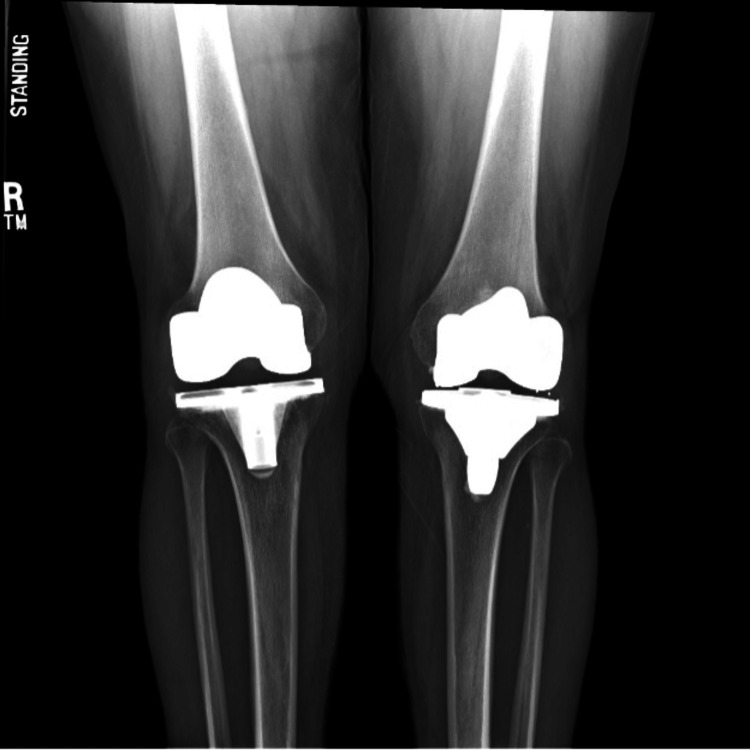
Bilateral AP X-ray of the knees.

**Figure 3:**
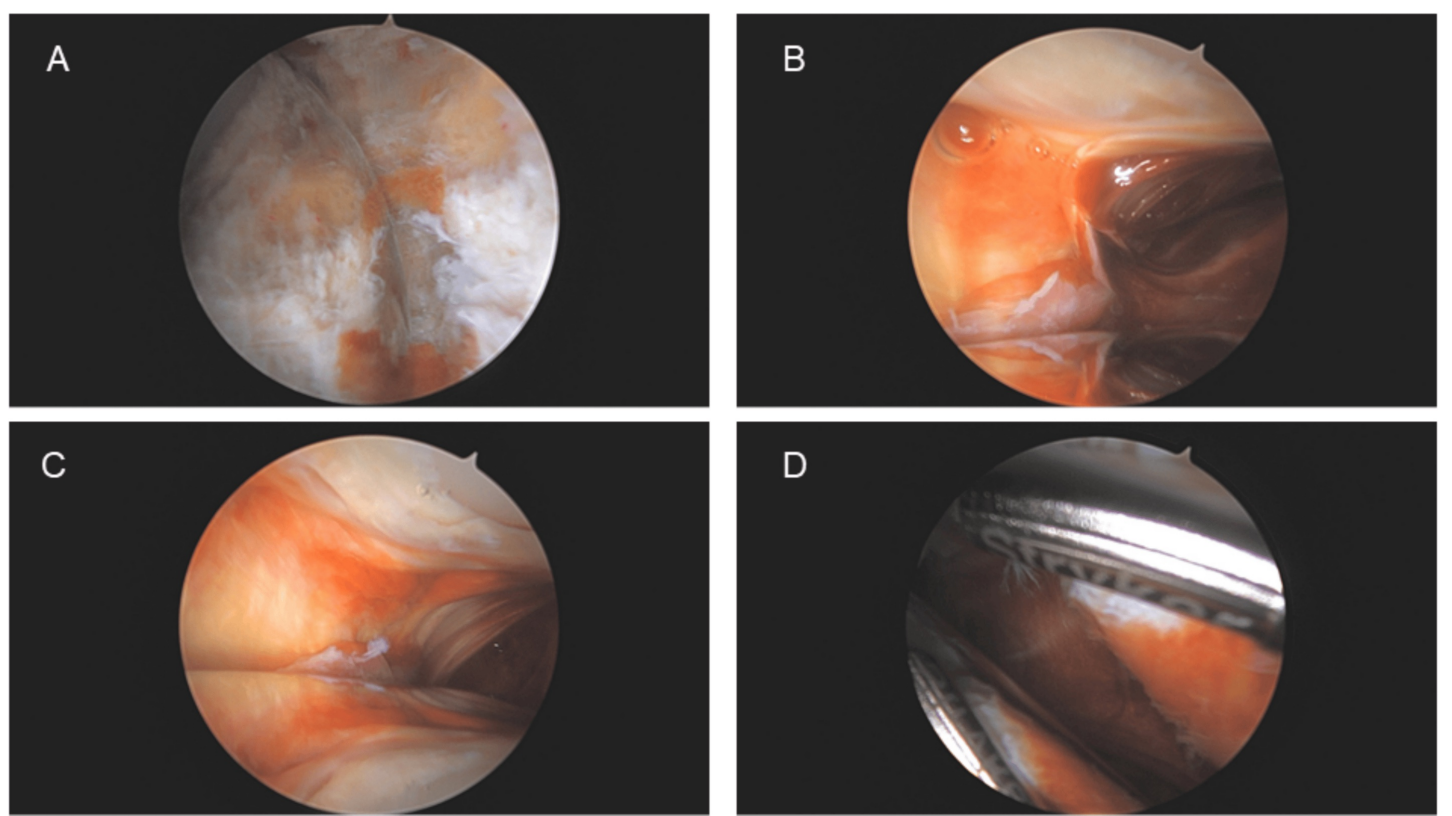
(A-D) Subsequent arthroscopy images showing diffuse pigmented villonodular synovitis.

Twenty-eight months after her knee arthroplasty and 15 months after her initial arthroscopy, the patient returned to the clinic with complaints of pain, restricted ROM, and crepitus in the right knee of atraumatic onset. On examination, a moderate effusion, pain, and crepitus with ROM were observed. Radiographs demonstrated stable and structurally intact TKA components with no signs of loosening (Figure 2). Due to the recurrence of symptoms, the patient was taken back to the operating room for a second diagnostic arthroscopy, which revealed fibrous nodules of scar tissue and extensive synovitis (Figure 3). The diagnosis of PCS along with suspected PVNS was made. Arthroscopically, synovectomy and scar excision were performed using an arthroscopic shaver. The TKA components remained well-positioned, with no evidence of polyethylene wear.

**Figure 4:**
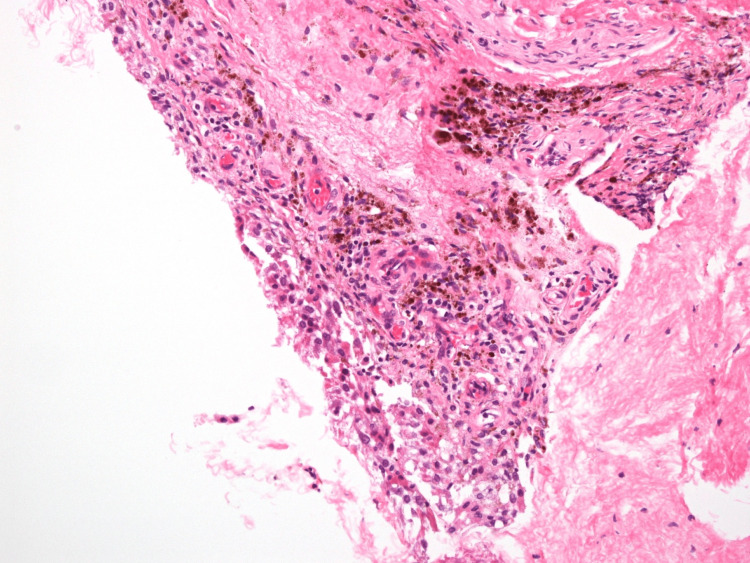
Light microscopy revealing fibrous nodules with foci of inflammation and hemosiderin deposition.

Histological analysis of the excised tissue confirmed the diagnosis of PVNS, showing fibrous nodules with foci of inflammation and hemosiderin deposition (Figure 4). Two weeks after the arthroscopic synovectomy, the patient was seen in the clinic, reporting mild discomfort but was otherwise progressing well.

## Discussion

D-PVNS is a benign yet inflammatory hyperproliferative synovial disorder that exhibits neoplastic mutations in 2%-16% of cases [3]. This progressive disease typically presents with non-specific symptoms, including pain and swelling, and less frequently with mechanical symptoms and limited ROM [3]. Synovectomy is the primary treatment modality for PVNS; however, the optimal approach remains to be determined and may vary based on tumor size and location [4,17].

Recurrence rates in cases of D-PVNS can reach up to 50%, with the open versus arthroscopic approach showing mixed results. There are differing opinions among experts regarding the approach; some recommended open synovectomy for better exposure, while others lean toward arthroscopic resection due to lower morbidity and superior functional outcomes. Several centers have adopted a combined approach, using anterior arthroscopic and open posterior approaches, as it allows for more extensive debridement [4,17]. For instance, Colman et al. demonstrated significantly lower recurrence rates in patients treated with a combined approach compared to either all-arthroscopic or all-open approaches [18]. However, Kerschner et al. found no statistical difference in recurrence rates or functional outcomes between the combined and arthroscopic approaches [19].

It is also worth mentioning that for patients who are not surgical candidates, several systemic therapies targeting the CSF1 pathway have been evaluated. Among these, pexidartinib, a CSF1 receptor inhibitor, has received approval from the Food and Drug Administration (FDA) for the treatment of PVNS. Studies show that pexidartinib provides improved functional outcomes and demonstrates an excellent overall response rate [4,17].

In our case, we report a unique occurrence of D-PVNS along with PCS following TKA and subsequent arthroscopy. While there is no established link between PVNS and TKA, a few cases in the literature describe PVNS occurring in patients with no known prior history of the disease [10-16].

For example, Kia et al. [15] reported a case of L-PVNS four years after TKA, presenting as recurrent clinical hemarthrosis without synovitis or hemarthrosis identified during arthroscopy one year after surgery. The authors proposed trauma as the likely trigger for PVNS in that patient. The spontaneous occurrence of PVNS has also been described in the literature [12]. On the other hand, Bunting et al. [11] presented a case of L-PVNS one year after bilateral TKA, suggesting that the development of PVNS may have been due to the surgical procedure itself. Although the exact cause of D-PVNS in our patient is uncertain, it is possible that trauma from scar excision for PCS may have triggered the condition [8], given that it appeared approximately 15 months after the procedure and there were no signs of synovitis or hemarthrosis during the initial arthroscopy.

No known association between PCS and PVNS has been documented in the literature to date, and it is unknown which condition developed first. However, given that PVNS is a slowly progressive chronic disease, this rare occurrence raises the question of whether the inflammatory microenvironment associated with PVNS [4] may have contributed to the recurrence of PCS in this case.

Overall, this case highlights the importance of considering PVNS in the differential diagnosis of patients presenting with non-specific symptoms or when a less aggressive disease is suspected following TKA. Additional research is required to delve into the connection, between PVNS and TKA and the presence of PCS, in instances.

## Conclusions

We present the rare occurrence of D-PVNS developing two and half years following TKA, alongside PCS. The possible etiology of this case may be related to surgical trauma from scar excision for PCS treatment. While no link between PVNS and PCS has been established, it is likely that the inflammatory environment could have triggered the recurrence of PCS. This case adds to the limited reports in the literature and underscores the importance of considering PVNS in the differential diagnosis of complications following TKA.

## References

[1] Kim SJ, Shin SJ, Choi NH, Choo ET (2000). Arthroscopic treatment for localized pigmented villonodular synovitis of the knee. Clin Orthop Relat Res.

[2] Mankin H, Trahan C, Hornicek F (2011). Pigmented villonodular synovitis of joints. J Surg Oncol.

[3] Kager M, Kager R, Fałek P (2022). Tenosynovial giant cell tumor. Folia Med Cracov.

[4] van der Heijden L, Spierenburg G, Kendal JK, Bernthal NM, van de Sande MA (2023). Multimodal management of tenosynovial giant cell tumors (TGCT) in the landscape of new druggable targets. J Surg Oncol.

[5] Ehrenstein V, Andersen SL, Qazi I, Sankar N, Pedersen AB, Sikorski R, Acquavella JF (2017). Tenosynovial giant cell tumor: incidence, prevalence, patient characteristics, and recurrence. A registry-based cohort study in Denmark. J Rheumatol.

[6] Rao AS, Vigorita VJ (1984). Pigmented villonodular synovitis (giant-cell tumor of the tendon sheath and synovial membrane). A review of eighty-one cases. J Bone Joint Surg Am.

[7] Hantes ME, Basdekis GK, Zibis AH, Karantanas AH, Malizos KN (2005). Localized pigmented villonodular synovitis in the anteromedial compartment of the knee associated with cartilage lesions of the medial femoral condyle: report of a case and review of the literature. Knee Surg Sports Traumatol Arthrosc.

[8] Panciera A, Colangelo A, Di Martino A (2024). Total knee arthroplasty in pigmented villonodular synovitis osteoarthritis: a systematic review of literature. Musculoskelet Surg.

[9] Sequeira SB, Scott J, Novicoff W, Cui Q (2020). Systematic review of the etiology behind patellar clunk syndrome. World J Orthop.

[10] Ballard WT, Clark CR, Callaghan JJ (1993). Recurrent spontaneous hemarthrosis nine years after a total knee arthroplasty. A presentation with pigmented villonodular synovitis. J Bone Joint Surg Am.

[11] Bunting D, Kampa R, Pattison R (2007). An unusual case of pigmented villonodular synovitis after total knee arthroplasty. J Arthroplasty.

[12] Oni JK, Cavallo RJ (2011). A rare case of diffuse pigmented villonodular synovitis after total knee arthroplasty. J Arthroplasty.

[13] Chung BJ, Park YB (2011). Pigmented villonodular synovitis after TKA associated with tibial component loosening. Orthopedics.

[14] Camp CL, Yuan BJ, Wood AJ, Lewallen DG (2016). Pigmented villonodular synovitis diagnosed during revision total knee arthroplasty for flexion instability and patellar fracture. Knee.

[15] Kia C, O'Brien DF, Ziegler C, Pacheco R, Forouhar F, Williams V (2018). An unusual case of pigmented villonodular synovitis after total knee arthroplasty presenting with recurrent hemarthrosis. Arthroplast Today.

[16] Dedhia N, Zamata-Ovalle D, Johnson E, Schwechter E (2024). Localized tenosynovial giant cell tumor after total knee arthroplasty. Arthroplast Today.

[17] Bernthal NM, Ishmael CR, Burke ZD (2020). Management of pigmented villonodular synovitis (PVNS): an orthopedic surgeon's perspective. Curr Oncol Rep.

[18] Colman MW, Ye J, Weiss KR, Goodman MA, McGough RL 3rd (2013). Does combined open and arthroscopic synovectomy for diffuse PVNS of the knee improve recurrence rates?. Clin Orthop Relat Res.

[19] Kerschner A, King D, Vetter C (2021). Clinical outcomes of diffuse PVNS of the knee following arthroscopic complete synovectomy±posterior open resection. J Orthop.

